# Repeatability of a Short Food Frequency Questionnaire to Assess Calcium Intake in Older Australians

**DOI:** 10.4061/2010/905056

**Published:** 2010-06-07

**Authors:** Michelle Miller, Yee Chi Yeo, May Jiun Khor, Emma Clover, Anthea Magarey

**Affiliations:** Nutrition and Dietetics, Clinical and Molecular Medicine, Flinders University, GPO Box 2100, Adelaide SA 5001, Australia

## Abstract

*Objective*. To assess the repeatability of the validated Flinders Calcium Food Frequency Questionnaire (FFQ_CA_) for estimating dietary calcium intake in a sample of community dwelling older Australians. *Methods*. A test-retest repeatability study involving 100 subjects (≥65 years) living independently in metropolitan Adelaide, Australia. Estimates of daily calcium intake from the first (FFQ_CA1_) and repeated administration (FFQ_CA2_) were calculated from two versions (35-item and 15-item) of the FFQ_CA_. The intraclass correlation coefficient (ICC) was used to assess the repeatability. *Results*. Moderate and comparable ICC values (*r* = 0.5, *r* = 0.6) were found across the two versions of the FFQ_CA_. *Conclusion*. Both FFQ_CA_ versions demonstrated moderate repeatability, supporting the results of previous studies.

## 1. Introduction

Osteoporosis is a growing health issue in Australia [1]. It is characterized by low bone density and microarchitectural deterioration of bone tissue, with an increase in bone fragility and susceptibility to fractures, especially of the hip, spine, and wrist [1, 2]. Osteoporosis-induced fractures cause a great burden to society, with one in two Australian women and one in three Australian men over 60 years being likely to have an osteoporotic fracture and subsequent hospital admission every eight minutes [3, 4]. The absolute number of osteoporotic fractures is expected to increase dramatically by the year 2021 due to the increasing elderly population [3]. Furthermore, the health expenditure associated with the treatment of osteoporotic fractures and related complications is considerable, approximating $774 million annually in Australia [3, 4].

Inadequate calcium intake is a recognized risk factor for osteoporosis [5], with increased dietary calcium reducing the rate of bone loss and the risk of osteoporosis [2, 6]. However, despite numerous health promotion activities focused on bone health, the majority of older Australians fail to obtain adequate calcium intake [7]. In 1995, the National Nutrition Survey revealed that 645/1058 (61%) of women and 325/902 (36%) of men, aged 65 years and over, had calcium intakes below the recommended dietary intake (RDI) (800 mg for males and 1000 mg for females) [8]. In 2006, however, the RDI revised with the recommended daily calcium intake increased to 1000 mg (or 2 glasses of milk + 1 tub yoghurt + 1 cube of cheese) for males and 1300 mg (or 2 glasses of milk + 2 tubs yoghurt + 1 cube of cheese) for females. Therefore, it is predicted that a greater proportion of older Australians have an inadequate calcium intake based on these new recommendations [9]. By identifying individuals who are at risk of osteoporosis through inadequate calcium intake, early treatment or prevention of osteoporosis is possible [10].

Identification of inadequate calcium intake is complex. One method commonly used is the Food Frequency Questionnaire (FFQ) due to the simple format, cost efficiency, ability to be self-administered, and ability to provide insight regarding long-term and usual dietary intake [5, 11]. Due to the implications of low calcium intake, three calcium specific FFQs have previously been developed in Australia [7, 9, 12]. Two of these however have not been validated in older adults [7, 12] and none have been evaluated for repeatability [7, 9, 12].

The FFQ specifically validated in older Australians is the Flinders Calcium FFQ (FFQ_CA_). It was designed specifically for use among older Australians and has been validated according to the revised recommendations [9]. The relative validation of the FFQ was performed on 102 (67F) aged 65 years or greater using data collected between 2002 and 2006. Calcium intake determined from each version (15 item and 35 item) of the FFQ was tested for agreement against average intake from four nonconsecutive weighed food records. Mean bias between the food record and the 35-item and 15-item FFQ was 5 mg and 28 mg, respectively. Levels of sensitivity and specificity for identifying older adults at risk of not achieving estimated average requirements were comparable to other FFQs designed to evaluate calcium intake. The aims of the present study were to evaluate the repeatability of the Flinders FFQ_CA_ in a sample of older Australians and examine whether shortening the 35-item FFQ_CA_ to a 15-item version would affect its repeatability.

## 2. Methods

### 2.1. Subjects

Male and female participants of concurrent observational studies undertaken by the authors of this study, in addition to subjects from Adelaide metropolitan retirement villages, were recruited via mail. Inclusion criteria were that subjects were 65 years and over, living independently, and able to independently complete a FFQ. A target of 100 participants (50 female and 50 male) was set. This sample size was estimated using the methodology proposed by Walter et al. (1998) [13] for approximating the number of subjects required for test-retest reliability. Assuming 0.4 to be the minimally acceptable level of reliability but hypothesising reliability of 0.7 with administration of the questionnaire on 2 occasions, a sample size of 33 is considered appropriate (*P* < .05, 80% power). To account for attrition between the first and second administration, the sample size target was inflated to 50 females and 50 males (total *n* = 100). Ethical approval for this study was granted by the Flinders University Social and Behavioural Research Ethics Committee, Adelaide, Australia.

### 2.2. Data Collection Procedure

A home visit was arranged for interested subjects, at which time they received comprehensive verbal and written information on the study. Subsequently, informed consent was obtained and information on participant medical history, list of current medications, use of vitamin and mineral supplements, alcohol intake, and smoking habits were collected. Subjects' weight (without shoes) was measured (nearest 0.1 kg) using a digital scale (Tanita BF-681, Japan-body fat not reported), and knee-height (on the left leg), which was later converted to a standing height [14], was measured (nearest 0.1 cm) using a knee-height calliper (Ross Laboratories, Washington, USA). The FFQ_CA_ was subsequently completed. The FFQ_CA_ is a single questionnaire containing a total of 36 items, 21 items unique to the 35-item FFQ instrument, 1 item unique to the 15-item FFQ instrument (i.e., one item assessing total daily milk intake replaced 21 items referring to details of daily milk intake from the 35-item version) and 14 items that were included in both versions of the FFQ instruments. It is estimated that each questionnaire took 10 minutes to complete. Additional explanation or clarification was provided by the assistant when required. The assistant also reviewed the FFQ_CA_ to ensure clarity and completeness.

After completing the first FFQ_CA_ (FFQ_CA1_), a second identical questionnaire (FFQ_CA2_) together with written instructions and a reply paid envelope were sent to each subject to complete and return. Participants also received a reminder phone call on the date the FFQ_CA2_ was due (2 weeks post FFQ_CA1_) to ensure compliance and provide assistance if needed. Each FFQ targeted usual intake of calcium over the preceding 12 months.

### 2.3. Calculation of Calcium Intake

*Foodworks* (Foodworks Professional Edition 1998–2005 Xyris Software Australia, data: 2004 and 2006) and *Nutritional Values of Australian Foods* (Data: 2002) [15], a published document of food composition tables, both of which use the same food composition data [16], were used to determine the calcium content (mg/100 g) for each food item in the FFQ_CA_. The calcium content in the nutritional information panel of each item was used if subjects provided specific product names. 

The total daily calcium intake derived from both FFQ_CA1_ and FFQ_CA2_ was calculated for each subject, using Microsoft Office Excel (Microsoft Corporation, 2007). This was done in two stages, creating a 35-item and a 15-item FFQ_CA_. The calcium intake obtained from vitamin and mineral supplements was calculated separately. If the calcium content of multivitamin and mineral supplements was not specified, the average calcium content (73 mg/dose) from four most commonly used supplements among participants was used.

### 2.4. Data Analysis

Mean ± standard deviation (SD) (if the data were normally distributed) or median + inter quartile range (IQR) (if the data were not normally distributed) were calculated for each subject's age, weight (kg), height (cm), Body Mass Index (BMI) (calculated as weight (kg)/height (m^2^)), and total estimated calcium intake. To account for nonnormality, calcium intake was log transformed before the repeatability test was performed for both the 35-item and 15-item FFQ versions. The intraclass correlation coefficient (ICC) was used to test the repeatability of total daily calcium intake calculated from the FFQ_CAs_. All statistical analyses were performed using SPSS 14.0 for Windows (SPSS Inc., Chicago, Illinois, USA).

## 3. Results

### 3.1. Subjects

Baseline characteristics of participants are shown in Table 1. Of all participants (*n* = 100), 71% were female. The mean (SD) age of subjects was 77 (±6) years. The mean (SD) weight and height were 71.1 kg (±14.7) and 162.2 cm (±8.1), respectively. Mean (SD) BMI was 26.9 kg/m^2^ (±4.9) with 50% of the sample having a BMI within the acceptable range (22–27 kg/m^2^) for the elderly [17]. Seventy-nine subjects reported at least one underlying clinical condition including hypertension (*n* = 22), arthritis (*n* = 19), osteoporosis (*n* = 10), and type 2 diabetes (*n* = 10). Sixty-four subjects reported usual intake of vitamin and mineral supplements, yet only 36 of them consumed a calcium supplement. The median (IQR) intake of calcium from supplements in the 38 participants reporting consumption on first administration of the FFQ was 600 mg (230–600).

### 3.2. Estimated Calcium Intake from the 35- and 15-Item Versions of Repeated *FFQ*_*CA*_

The mean total estimated daily calcium intake from food and beverage items (excluding supplements) did not vary significantly between FFQ_CA1_ and FFQ_CA2_ for either the 35-item or 15-item FFQ_CA_ version (difference of 7.3 mg and 13.3 mg, resp.).

### 3.3. Repeatability Test

The ICC was 0.5 (95%CI 0.27–0.59) for the 35-item FFQ_CA_ and 0.6 (95%CI 0.48–0.73) for the 15-item FFQ_CA_ (Table 2).

## 4. Discussion

This is the first study to evaluate the repeatability of a validated FFQ for estimating calcium intake, in a sample of community dwelling older Australians (≥65 years). This study was strengthened by recruiting a sample size estimated to detect meaningful values for ICC, particularly for females. Additionally, the FFQ was short and simple, thus reducing random response error due to respondent burden [18]. 

This study showed that there was little variance in repeatability for both the 35-item and 15-item FFQ_CA_, indicating that a high level of reliance can be expected on a single administration of the FFQ_CA_ in older Australians. The slight variances between repeated FFQ administrations were most likely due to the “true” difference contributed by subject variance, instead of measurement errors or within-subject variance [11]. The difference of 7.3 mg for the 15-item FFQ_CA_ and 13.3 mg for the 35-item FFQ_CA_ correspond to approximately 8 mL and 15 mL of regular milk, respectively, and are thus negligible in practice. The repeatability results presented here are slightly different to those of two similar studies. Lazarus et al.'s [19] study using 62 elderly participants (≥65 years) reported an ICC value of 0.72, while Pietinen et al. [20] studied 121 men (≥55 years) and reported an ICC value of 0.70. While these figures suggest better repeatability than that shown by our study, the different methodologies make direct comparisons inappropriate. Both studies administered FFQs for multinutrient intake and extracted individual nutrients for analysis, including calcium. In addition, there was more investment in prompting participants to respond than in the present study.

The comparable repeatability between the two FFQ_CA_s indicates that the 15-item FFQ_CA_ can be used in place of the longer version, thus it would be particularly useful as a clinical screening tool. However, the 35-item questionnaire also has value as it provides more information on sources of calcium in the diet. Furthermore, Clover et al. (2007) [21] noted that the short FFQ_CA_ did not assess those with very high calcium intakes with as much accuracy as the 35-item FFQ_CA_. The authors would recommend the 35-item FFQ_CA_ where a more detailed assessment of calcium intake is required.

It should be acknowledged that responses from elderly participants may be limited by problems due to aging, such as failing memory or cognition [22]. However, the moderate level of repeatability shown in this study suggests that any negative effect of ageing on repeatability had been offset by an inherently more stable pattern of food purchase and meal preparation, and thus, more consistent and routine daily dietary consumption patterns among older adults were supported by previous literature [19, 22]. Consequently, this tool may have limited error related to poor recall ability associated with older adults [19]. It should also be acknowledged that there were not equal numbers of females and males recruited for participation in this study. While the proportion was similar to the population, it is possible that with a greater number of males there could have been a change in the findings of this study, particularly given the recruited number of males fell slightly short of the estimated sample size requirement. A recent Canadian study found that women were more likely to consume dairy products than men [23]. This may be because women are perceived to be more likely than men to engage in healthier eating patterns and to adopt lifestyle changes to address such issues as weight management and osteoporosis prevention.

Finally, it must be acknowledged that responding to a single questionnaire containing all of the items of interest may result in different conclusions than administering two questionnaires, one version being the 15-item instrument and the other being the 35-item instrument. The authors chose not to do the latter due to the replication of items between the two instruments and the additional participant burden that would be required.

## 5. Conclusion

The comparable repeatability between FFQ_CA1_ and FFQ_CA2_ seen for the 15-item and 35-item FFQ_CA_ demonstrates that the 15-item FFQ, as it requires less time and effort to complete thus increasing accuracy, is the preferred tool to use in clinical and research settings for older Australians. Therefore, the Flinders Calcium FFQ could be used as a rapid screening tool to identify older Australians who are at risk of inadequate calcium intake in health care settings and thus could allow for earlier prevention and intervention to reduce the incidence of osteoporosis.

## Figures and Tables

**Table 1:** Baseline characteristics of study participants.

Baseline characteristics	Participants, *n*	
	Male (*n* = 29)	Female (*n* = 71)
Age		
65–74	7	33
75–84	17	35
≥85	5	3

BMI (kg/m^2^)		
<22	1	7
22–27	20	30
>27	8	34

Marital status		
Married	17	20
Never married/ single	0	7
Divorced/separated	3	10
Widowed	9	34

Takes a calcium supplement		
Yes	4	32
No	25	39

Consumes Alcohol		
Yes	17	31
No	8	35
Occasionally	4	5

Smoker		
Yes	0	2
No	29	69

**Table 2:** Median (IQR) estimated daily total calcium intake, intraclass correlation coefficients (ICC), and confidence interval (95% CI) of repeated administration of the 35-item and 15-item Flinders Calcium Food Frequency Questionnaire (*FFQ*_*CA*_) in study participants (≥65 years).

	Categories of items (number of items in each category)	Estimated daily total calcium intake (mg)		ICC (*r*)	95% CI	
		FFQ_CA1_ [Median (IQR)]	FFQ_CA2_ [Median (IQR)]		Lower	Upper
35 items	Milk-based beverages (7); dairy products including cheese, yoghurt and dairy-based dessert (13); bread and breakfast cereals (8); volume of milk added to beverages, breakfast cereals and porridge (5); type of milk used (1); type of bread used (1)	810 (576, 1100)	824 (589, 1136)	0.5	0.27	0.59

15 items	As original 35-item FFQ with *exclusion* of bread & breakfast cereals (8); volume of milk added to breakfast cereals and porridge (2); type of bread used (1). Replace volume of milk added to beverages (3) and all milk-based beverages (7) with single item in estimation of overall daily milk consumption	728 (485, 1019)	685 (447, 1019)	0.6	0.48	0.73
